# Impact of maternal obesity and mode of delivery on the newborn skin and oral microbiomes

**DOI:** 10.1099/jmm.0.002000

**Published:** 2025-04-10

**Authors:** Allison Seifert, Kelly Ingram, Embelle Ngalame Eko, Jaclyn Nunziato, Monica Ahrens, Brittany R. Howell

**Affiliations:** 1 Virginia Tech Carilion School of Medicine, Roanoke, VA, USA; 2 Carilion Clinic, Roanoke, VA, USA; 3 Department of Statistics at Virginia Tech, Blacksburg, VA, USA; 4 Fralin Biomedical Research Institute at VTC, Roanoke, VA, USA; 5 Department of Human Development and Family Science, Virginia Tech, Blacksburg, VA, USA

**Keywords:** immune imprinting, newborn microbiome, newborn seeding, obesity, oral microbiome, pregnancy, skin microbiome

## Abstract

**Introduction.** Previous studies have shown vast differences in the skin and oral microbiomes of newborns based on delivery method [Caesarean section (C-section) vs vaginal]. Exposure to or absence of certain bacteria during delivery can impact the neonate’s future susceptibility to infections, allergies or autoimmunity by altering immune functions. Few studies have focused on the impact of maternal obesity on the variations of newborn skin and oral microbiomes. Obese pregnant women typically have a higher vaginal microbiome diversity, and their pregnancies are at higher risk for adverse outcomes and complications.

**Hypothesis.** We hypothesized that the skin and oral microbiomes of newborns born to obese mothers would include more diverse, potentially pathogenic bacteria and that the skin and oral microbiome in C-section delivered newborns would be less diverse than vaginally delivered newborns.

**Aim.** We aim to begin to establish maternal obesity and mode of delivery as factors contributing to increased risk for negative newborn outcomes through impacts on newborn bacterial dysbiosis.

**Methodology.** A skin swab was collected immediately following delivery of 39 newborns from 13 healthy weight body mass index (BMI 18.50–24.99), 11 overweight (BMI 25.0–29.99) and 15 obese (BMI ≥30.00) pregnant participants. An oral swab was collected immediately following delivery for 38 of these newborns from 13 healthy weight, 10 overweight and 15 obese pregnant participants. Bacterial genera were identified via 16S rRNA amplicon sequencing.

**Results.** The newborn skin microbiome was comprised of typical skin bacteria (i.e. *Corynebacterium*). Newborns of obese participants had a higher relative abundance of *Peptoniphilus* in their skin microbiome compared to newborns of healthy weight participants (*P*=0.007). Neonates born via C-section had a higher relative abundance of *Ureaplasma* in their oral microbiome compared to neonates delivered vaginally (*P*=0.046).

**Conclusion.** We identified differences in the newborn skin and oral microbiomes based on pre-pregnancy BMI and method of delivery. These differences could be linked to an increased risk of allergies, autoimmune disease and infections. Future longitudinal studies will be crucial in determining the long-term impact of these specific genera on newborn outcomes. Understanding these connections could lead to targeted interventions that reduce the risk of adverse outcomes and improve overall health trajectory.

## Introduction

The infant microbiome is derived from both maternal and environmental sources during and after delivery. Because the intrauterine environment has traditionally been considered sterile, it has been suggested that the newborn acquires its first microflora of the skin, nose, mouth and conjunctiva from the mother’s birth canal during vaginal delivery [1]. More recent evidence also shows that there could be additional intrauterine microbial factors at play during pregnancy, including colonization of the placental basal plate by diverse bacteria [2]. These microbial exposures, including intrauterine exposures and exposures during and immediately after birth, are thought to drive postnatal immune development and have been linked to postnatal outcomes, such as the development of allergies and autoimmune disease [2,3].

Immune imprinting involves the early relationships between commensal microbes and the developing immune system, which fine-tunes immune cells to respond appropriately to pathogens and tolerate self or harmless environmental antigens [4]. Exposure to or the absence of certain bacteria during delivery can impact future susceptibility to disease, allergies or autoimmunity by altering immune functions [5]. For example, previous studies have shown that the risk of being diagnosed with allergic rhinoconjunctivitis was significantly higher in children born by Caesarean section (C-section) than in those delivered vaginally [6]. Additionally, a retrospective review of two million children from the Danish Medical Birth Registry and the Danish National Patient Registry showed that children born by C-section had a significantly increased risk of asthma, systemic connective tissue disorders, juvenile arthritis, inflammatory bowel disease, immune deficiencies and leukaemia [7]. These differences in outcomes could be due to differences in microbial exposure at birth. Moreover, lack of developed host defence mechanisms in infants makes them vulnerable to opportunistic infections from both commensal and pathogenic bacteria shortly after birth [8]. In addition to this immune imprinting effect, maternal health during pregnancy can also impact infant development.

Obesity during pregnancy has been associated with both maternal and neonatal complications. Associated maternal complications include insulin resistance, hypertension, venous thromboembolism and anaesthesia-related complications [9]. Neonatal complications of obese mothers include increased risk of preterm birth, miscarriage, neural tube defects, omphalocele, cardiac defects and macrosomia [9]. Of particular relevance for the current investigation, neonates of obese mothers have been shown to be more prone to neonatal sepsis within 72 h of birth [10], and more likely to die of infections for up to 18 years after birth [11]. Given this connection between offspring infection and maternal obesity, and the links between early microbial exposures and later immune function, it is likely that maternal obesity may be impacting infants through alterations in the typical infant microbiomes.

Previous studies of the neonatal oral microbiome identified several species of bacteria, both commensal and pathogenic, commonly found in the oral cavity of neonates [12,13]. Notably, vaginally delivered neonates tended to develop oral microbiota that were similar to the vaginal microbiome of the mothers, dominated by *Lactobacillus, Prevotella* and *Sneathia*, while those delivered by C-section developed microbiota that were more similar to the mothers’ skin communities, predominantly *Staphylococcus* and *Corynebacterium* [14]. The skin microbiome of newborns has been shown to be vastly different based on delivery method (C-section vs vaginal) with a wider variety of vaginal microbes present in vaginally delivered newborns compared to C-section delivered newborns [4]. Vaginal delivery has evolved to expose the newborn to the gut and vaginal flora of the mother, providing a pathway for newborn microbiome seeding, but also a pathway for potentially pathogenic bacteria to colonize the baby shortly after birth. Of relevance for this study, obese women do show alterations in their vaginal microbiomes.

Healthy vaginal microbiota is associated with a high prevalence of *Lactobacillus*, which helps to maintain an acidic pH and prevent the overgrowth of harmful bacteria [15]. Obese pregnant women typically have a higher vaginal microbiome diversity that includes potentially pathogenic bacteria, which can also be transferred to their newborns [16]. Most studies concerning the vaginal microbiome in pregnancy study only healthy weight populations. Thus, there is a lack of vaginal microbiome data of pregnant overweight or obese populations despite growing evidence of microbiome differences among weight groups outside of pregnancy [17]. There are known differences in the non-pregnant vaginal microbiome between weight groups, including higher bacterial diversity and lower *Lactobacillus* dominance in those with higher body mass index (BMI) [18], with these differences potentially continuing during pregnancy. Differences in vaginal microbiota may ultimately impact newborn microbiome seeding and subsequent immune imprinting; however, there is a paucity of data addressing if maternal pre-pregnancy BMI influences their newborn’s skin and oral microbiota, or if these differences impact birth outcomes.

Post-natal complications, such as neonatal sepsis, are more common in overweight and obese women [10]. If maternal BMI-related alterations in newborn microbiome seeding play a role, and the skin and oral microbiota can be assessed post-natally and associated with increased risk of adverse post-natal outcomes, these measures could be used to prospectively identify neonates who are at a greater risk for complications. We hypothesized that both the skin and oral microbiomes of newborns born to obese mothers would include more diverse, potentially pathogenic bacteria. Additionally, we hypothesized that both the skin and oral microbiomes in C-section delivered newborns would be less diverse than vaginally delivered newborns. With this information, skin and oral microbiota screenings could then be used as tools to determine infant risk for post-natal complications, thus allowing for earlier intervention and prevention of poor outcomes.

## Methods

### Study design

As part of a larger study, a total of 72 pregnant participants were recruited and enrolled in their first trimester of pregnancy [19]. Participants were recruited at Carilion Clinic Obstetrics and Gynecology clinics in Roanoke, VA. Participants were divided into three groups based on pre-pregnancy BMI (according to electronic health records) to include 23 healthy weight (BMI 18.50–24.99), 22 overweight (BMI 25.00–29.99) and 22 obese (BMI ≥30.00) participants. Participants were eligible for enrolment if: (1) they were between the ages of 18 and 45, (2) they had a confirmed pregnancy, (3) they planned to attend all their scheduled prenatal visits and (4) they planned to have a hospital delivery. Participants were excluded if: (1) they had a history of diabetes or hypertension, (2) they had a previous C-section, (3) they planned to have a scheduled C-section, (4) they had contraindications to vaginal delivery (e.g., severe previous vaginal tearing) or (5) they used antibiotics or probiotics within the past 3 months of study screening. We collected swabs from 39 of their newborns at the time of delivery (39 skin and 38 oral swabs). Skin swabs were collected from newborns born to 13 healthy weight, 11 overweight and 15 obese participants, and 38 oral swabs were collected from those same newborns born to 13 healthy weight, 10 overweight and 15 obese participants. The groups were balanced for factors that may influence outcomes, such as race and socioeconomic status assessed using annual household income and maternal education, through pre-screening and surveys (Table 1).

**Table 1.** Summary of participant demographics. Summary of age, race/ethnicity, highest level of education, yearly household income, delivery method and delivery term from participants whose newborn skin and oral swab samples were collected

Study group		Healthy weight	Overweight	Obese
Maternal age (Mean)		28.1	27	30.8
Maternal age (Range)		20–36	22–38	19–42
Race/Ethnicity	Caucasian/White	12 (92.3%)	9 (81.8%)	13 (86.7%)
	Black/African American	1 (7.7%)	0 (0%)	1 (6.7%)
	Asian	0 (0%)	1 (9.1%)	0 (0%)
	More than one race	0 (0%)	1 (9.1%)	1 (6.7%)
Highest level of education	High school diploma or GED	1 (7.7%)	0 (0%)	4 (26.7%)
	Some college	1 (7.7%)	1 (9.1%)	2 (13.3%)
	Associate's degree	1 (7.7%)	3 (27.3%)	2 (13.3%)
	Bachelor’s degree	6 (46.2%)	2 (18.2%)	1 (6.7%)
	Graduate degree	3 (23.1%)	4 (36.4%)	3 (20%)
	Unknown	1 (7.7%)	1 (9.1%)	3 (20%)
Yearly household income	Less than $29,999	1 (7.7%)	1 (9.1 %)	2 (13.3%)
	$30,000 to $99,999	5 (38.5%)	5 (45.5%)	7 (46.7%)
	$100,000 or above	6 (46.2%)	4 (36.4%)	3 (20%)
	Unknown	1 (7.7%)	1 (9.1%)	3 (20%)
Delivery method	Spontaneous vaginal delivery	11 (84.6%)	8 (72.7%)	12 (80%)
	Caesarean section (C-section)	2 (15.4%)	3 (27.3%)	3 (20%)
Delivery term	Preterm (<37 weeks)	0 (0%)	0 (0%)	1 (6.7%)
	Term (≥37 weeks)	13 (100%)	11 (100%)	14 (93.3%)

### Recruitment and retention

**Fig. 1.**
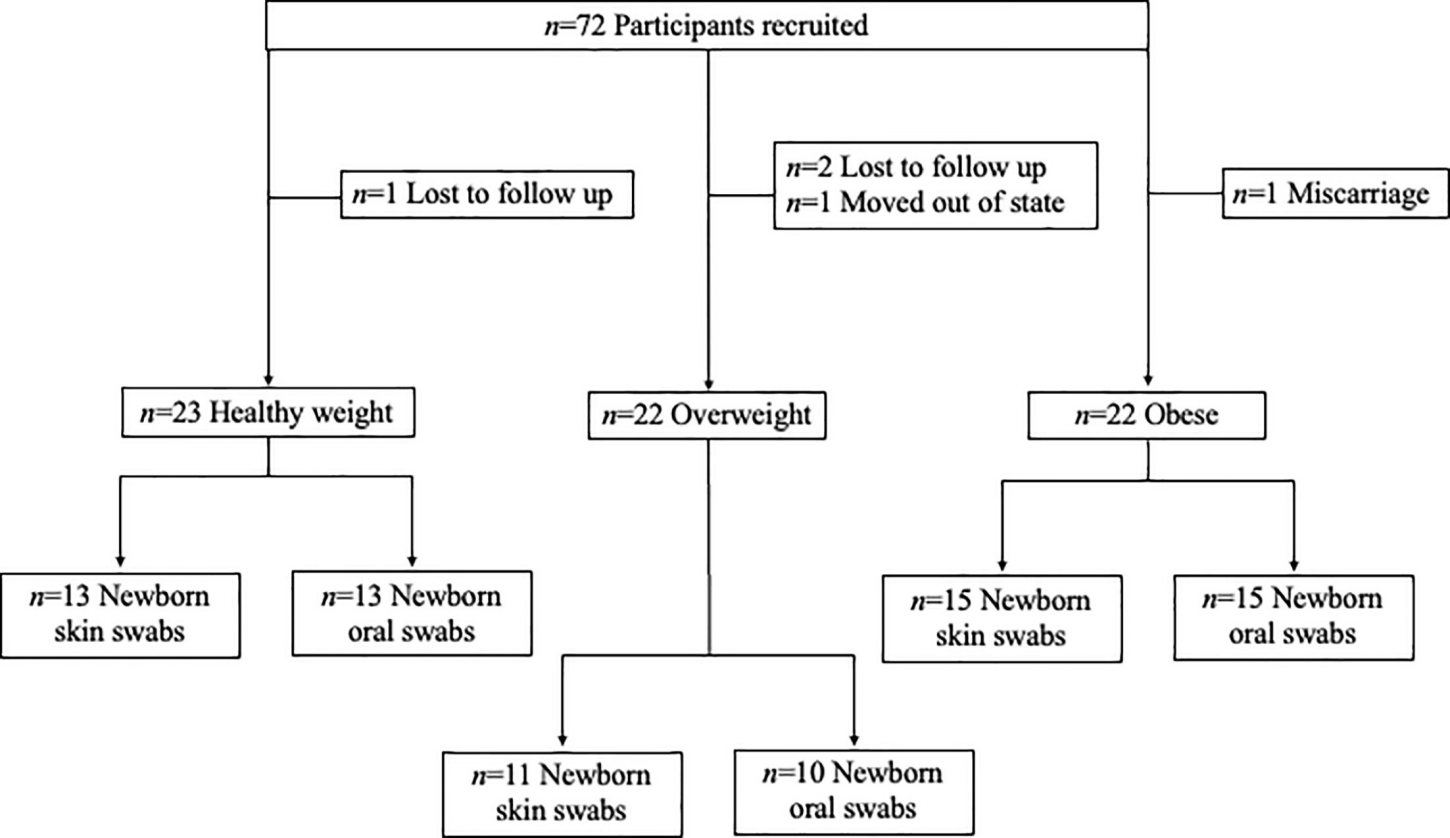
Participant recruitment. The total number of recruited and retained participants, including the total number of newborn samples collected from each study group (healthy weight, overweight and obese).

Participant recruitment occurred around the 8 week prenatal visit (Fig. 1). Each participant provided written informed consent approved by the Carilion Clinic Institutional Review Board. By the conclusion of the study, each participant received up to $125 in compensation on a reloadable debit card (ClinCard). At delivery, each participant was given a gift bag, valued at $50, with baby items to thank them for study completion.

### Participant data collection

Pertinent participant medical history was extracted from the electronic medical record (EMR), including age, pre-pregnancy BMI, diagnosed health conditions, medications, previous pregnancies and pregnancy outcomes or complications. All information from the EMR was confirmed with each participant prior to inclusion in the study. After delivery, information about the infant, including weeks of gestation, weight, APGAR (Appearance, Pulse, Grimace, Activity, and Respiration) score and mode of delivery, was extracted.

### Sample acquisition and processing

A vaginal swab sample was collected by the delivery team with the DNA Genotek OMNIgene Vaginal Microbiome DNA/RNA Collection Kit for microbiome sequencing. The vaginal wall was swabbed for 20 s, and the swab was placed into the provided container for stabilization and transport. C-section samples were collected almost exclusively from non-emergent C-sections because the participants who required an emergency C-section had more acute care needs and the swabs were not collected. Immediately following delivery, infant skin and oral swab samples were collected with the DNA Genotek OMNIgene Skin Microbiome DNA/RNA Collection Kit and DNA Genotek OMNIgene Oral Microbiome DNA/RNA Collection Kit for microbiome sequencing, respectively. The skin on the anterior neck was swabbed for 60 s, and the swab was placed into the provided container for stabilization and transport. The oral cavity was swabbed with a back-and-forth motion 10 times on each side of the mouth, and the swab was placed into the provided container for stabilization and transport. The vaginal, skin and oral samples were treated with Proteinase K and stored at −80 °C. Prior to DNA extraction, the samples were thawed and vortexed. DNA from skin samples was isolated using the ZymoBIOMICS DNA Microprep extraction kit according to the manufacturer’s protocol. DNA samples were quantified using the Qubit 4 fluorometer and Qubit™ dsDNA HS Assay Kit (Thermofisher Scientific). DNA from oral and vaginal samples was isolated using the QIAGEN DNeasy PowerSoil Pro Kit according to the manufacturer’s protocol. DNA samples were quantified using Qubit 4 fluorometer and Qubit™ dsDNA HS Assay Kit (Thermofisher Scientific). For skin samples, libraries were constructed by PCR amplification with primers covering the V1–V3 region (27F and 534R). Sequencing was performed on Illumina Miseq platform 2×300 bp. For vaginal and oral samples, libraries were constructed by PCR amplification with primers covering the V3–V4 region (341F and 805R). Sequencing was performed on the Illumina Miseq platform 2×250 bp. From the sequencing data generated by CosmosID, the relative abundance of each bacterial genus (*Lactobacillus*, etc.) and the alpha diversity (Chao1) of the samples were calculated.

### Statistical analysis

R Version 4.3.0 was used to generate figures for the average relative abundance of bacterial genera and pairwise relative abundance correlations [20,22]. A Wilcox rank sum test, generated by CosmosID-HUB Microbiome Comparative Analysis, was used to determine differences in alpha diversity and the relative abundance of bacterial genera. Spearman’s rank correlation (rho) was used to associate relative abundances across maternal and newborn microbiomes. Spearman’s rank correlation (rho) was first applied to combined maternal vaginal bacterial genera, combined newborn oral bacterial genera and combined newborn skin bacterial genera to explore key correlations of interest. Then, a correlation matrix was generated with pairwise correlations between all maternal genera and all newborn genera. For pairwise correlations, genera that had less than five participants (either maternal or newborn) with non-zero relative values of 0 were removed from the analysis. Participants who delivered via C-section and C-section delivered newborns were also excluded from pairwise analysis due to small sample size. A *P*-value of <0.05 was considered statistically significant. Due to our small sample size and the nature of this pilot study, we did not apply multiple comparisons corrections, nor were we able to investigate potential interaction effects.

## Results

### Infant skin microbiome

**Fig. 2.**
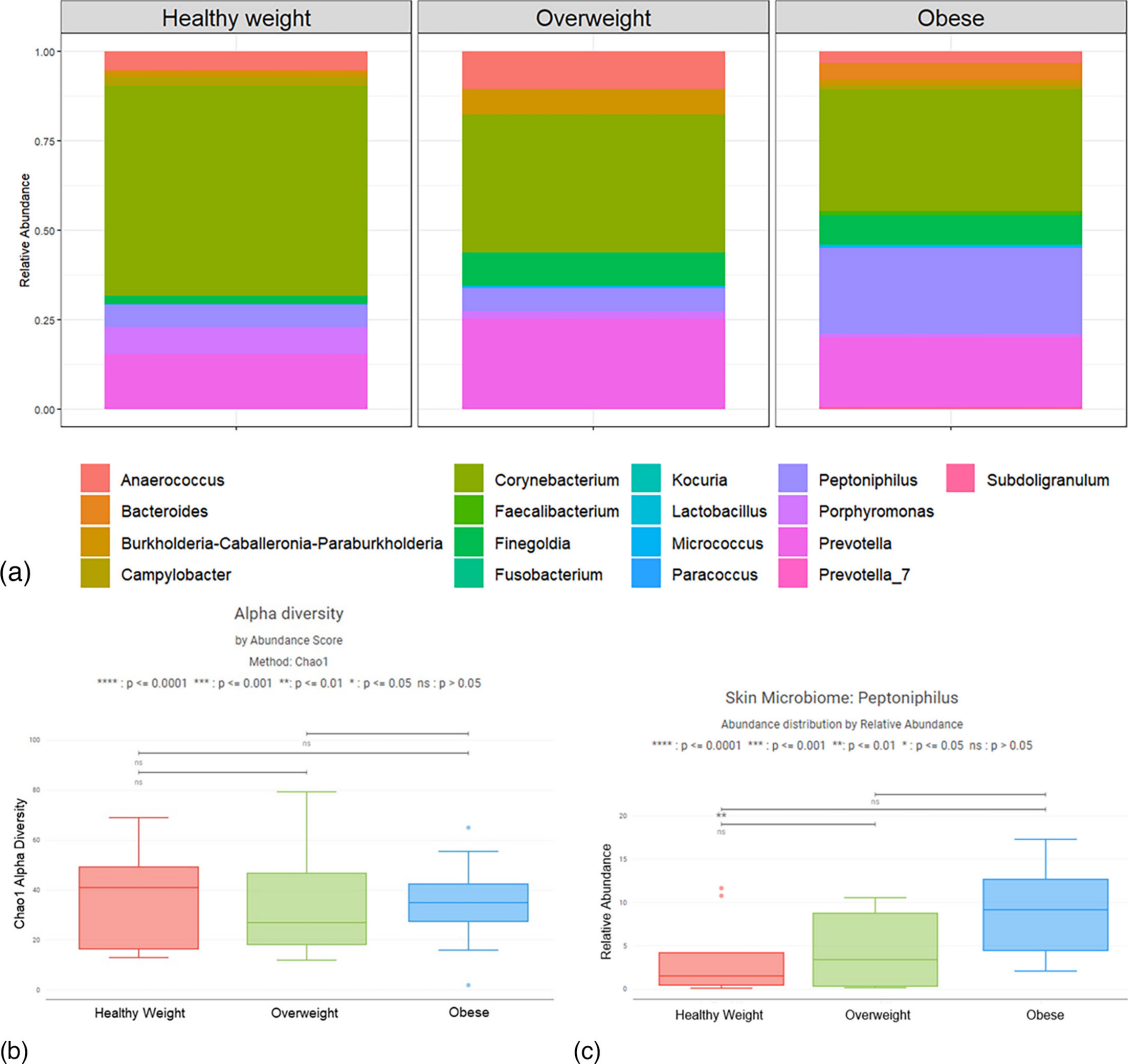
Relative Abundance of Newborn Skin Microbiota Across Maternal BMI Groups. (a) The average relative abundance of bacterial genera by BMI group at the time of delivery. (b) The median Chao1 alpha diversity of the skin microbiome of neonates from healthy weight, overweight and obese participants at the time of delivery was 41, 27 and 35, respectively, with no statistically significant differences between groups. (c) The median relative abundance of *Peptoniphilus* on the skin of newborns from obese participants was significantly higher compared to healthy weight participants at the time of delivery (*P*=0.007).

The infant skin microbiome was primarily comprised of *Corynebacterium* across all study groups (Table S1, available in the online Supplementary Material). Other prominent genera include *Finegoldia, Burkholderia-Caballeronia-Paraburkholderia, Campylobacter, Prevotella, Porphyromonas* and *Anaerococcus* (Fig. 2a). The median Chao1 alpha diversity, which estimates total bacterial richness, of the skin microbiome of neonates from healthy weight, overweight and obese participants at the time of delivery was 41, 27 and 35, respectively (Fig. 2b). There was no statistically significant difference in the Chao1 alpha diversity when comparing infants of healthy weight to overweight (*P*=0.706), healthy weight to obese (*P*=0.747) or overweight to obese participants (*P*=0.604). The relative abundance of *Peptoniphilus* species was higher in infants of obese participants compared to healthy weight participants (*P*=0.007), with medians of 1.5%, 3.4%, and 9.2% *Peptoniphilus* species in newborns born to healthy weight, overweight and obese participants, respectively (Fig. 2c). The relative abundance of *Peptoniphilus* when comparing newborns of healthy weight to overweight (*P*=0.909) and overweight to obese participants (*P*=0.055) was not statistically significant.

**Fig. 3.**
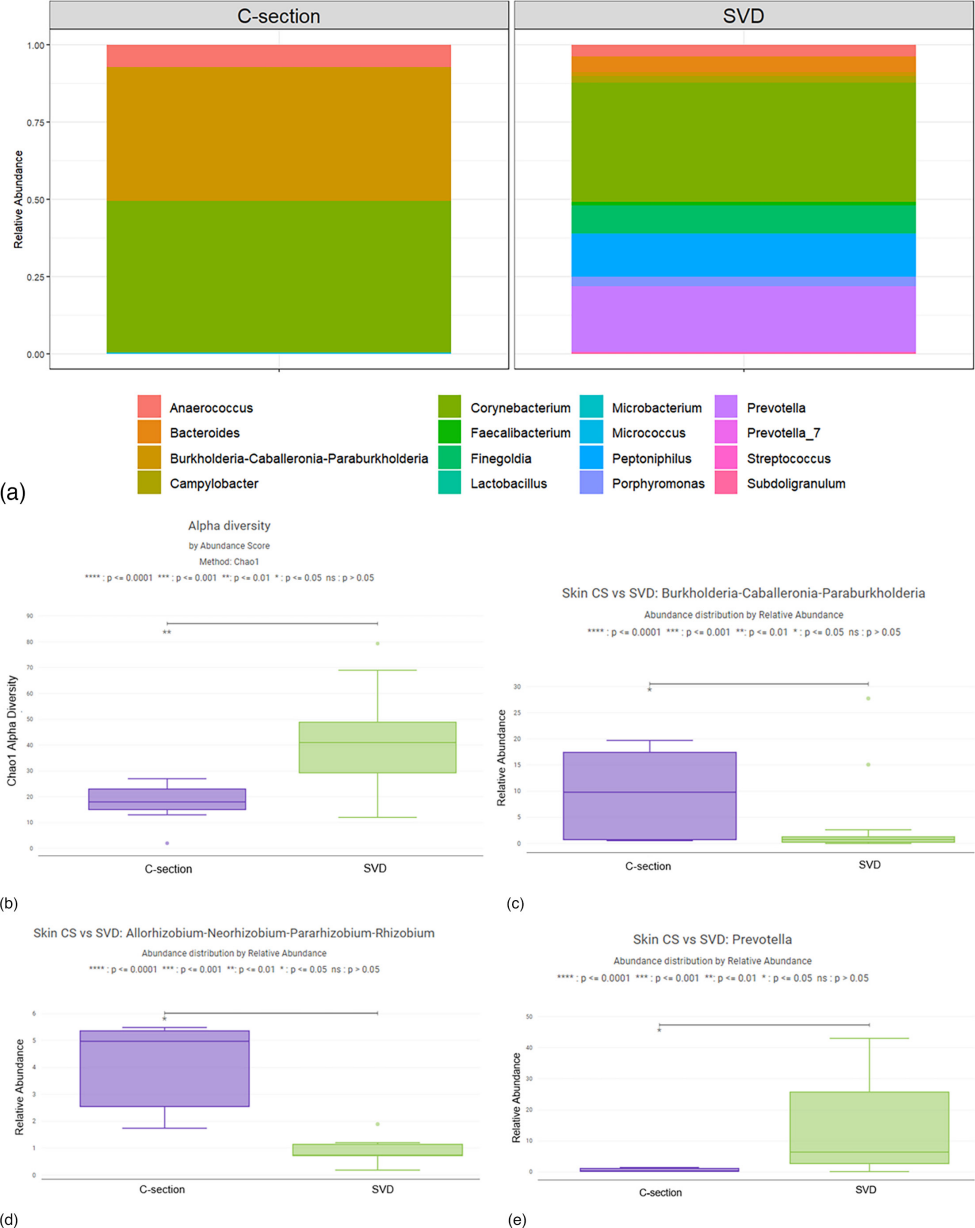
Relative Abundance of Newborn Skin Microbiota by Delivery Method. (a) The average relative abundance of bacterial genera by delivery method at time of delivery. (b) The Chao1 alpha diversity of skin microbiota was significantly higher in vaginally delivered newborns than C-section delivered newborns (*P*=0.002). (c) The relative abundance of *Burkholderia-Caballeronia-Paraburkholderia* on the skin of newborns delivered by C-section was significantly higher than those delivered vaginally (*P*=0.038). (d) The relative abundance of *Allorhizobium-Neorhizobium-Pararhizobium-Rhizobium* on the skin of newborns delivered by C-section was significantly higher than those delivered vaginally (*P*=0.03). (e) The relative abundance of *Prevotella* on the skin of newborns delivered vaginally was significantly higher than those delivered by C-section (*P*=0.039).

When comparing delivery method, C-section delivered neonates had a significantly lower median Chao1 alpha diversity than vaginally delivered neonates (*P*=0.002), with values of 18 and 41, respectively (Fig. 3a, b). C-section delivered newborns had a significantly increased relative abundance of *Burkholderia-Caballeronia-Paraburkholderia* compared to vaginally delivered infants (*P*=0.038) with medians of 9.8% and 0.8%, respectively (Fig. 3c). C-section delivered newborns also had an increased relative abundance of *Allorhizobium-Neorhizobium-Pararhizobium-Rhizobium* compared to vaginally delivered infants (*P*=0.03), with medians of 5.0% and 0.7%, respectively (Fig. 3d). Lastly, there was an increased relative abundance of *Prevotella* on the skin of vaginally delivered newborns compared to C-section delivered newborns (*P*=0.039) with medians of 6.4% and 0.2%, respectively (Fig. 3e).

### Infant oral microbiome

**Fig. 4.**
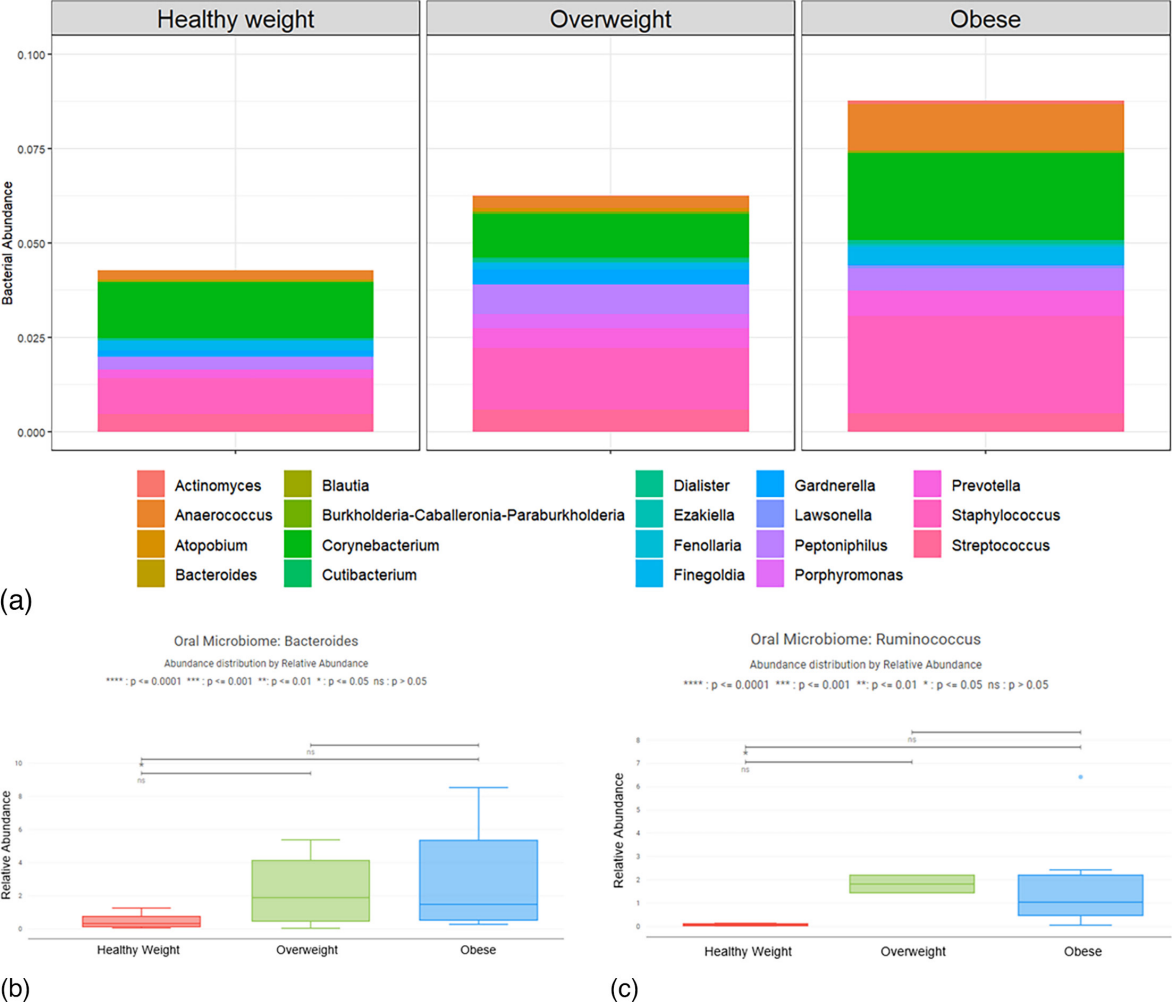
Relative Abundance of non-*Lactobacillus* Newborn Oral Microbiota Across Maternal BMI Groups. (a) The average relative abundance of non-*Lactobacillus* genera in the oral microbiota by group at time of delivery. (b) The median relative abundance of *Bacteroides* in the oral cavity of newborns from obese participants was significantly higher compared to healthy weight participants (*P*=0.048). (c) The median relative abundance of *Ruminococcus* in the oral cavity of newborns from obese participants was significantly higher compared to healthy weight participants (*P*=0.03).

The infant oral microbiome was primarily comprised of *Lactobacillus* across all study groups, which was removed from graphical representations to highlight other prominent genera, including *Corynebacterium, Actinomyces, Gardnerella, Staphylococcus, Streptococcus, Peptoniphilus* and *Anaerococcus* (Table S2, available in the online Supplementary Material, and Fig. 4a) The median Chao1 alpha diversity of the oral microbiome of neonates from healthy weight, overweight and obese participants at the time of delivery was 29, 27.5 and 39.33, respectively. There was no statistically significant difference in the Chao1 alpha diversity when comparing infants of healthy weight to overweight (*P*=0.852), healthy weight to obese (*P*=0.084) or overweight to obese participants (*P*=0.279). There was no statistically significant difference in the relative abundance of *Lactobacillus* when comparing infants of healthy weight to overweight (*P*=0.882), healthy weight to obese (*P*=0.995) or overweight to obese participants (*P*=0.921). The relative abundance of *Bacteroides* was higher in the oral cavities of newborns from obese participants compared to newborns from healthy weight participants (*P*=0.048), with medians of 0.3%, 1.9%, and 1.5% *Bacteroides* species from newborns born to healthy weight, overweight and obese participants, respectively (Fig. 4b). Additionally, the relative abundance of *Ruminococcus* was higher in the oral cavities of newborns from obese participants compared to newborns from healthy weight participants (*P*=0.03), with medians of 0.04%, 1.82%, and 1.04% *Ruminococcus* species from newborns born to healthy weight, overweight and obese participants, respectively (Fig. 4c).

**Fig. 5.**
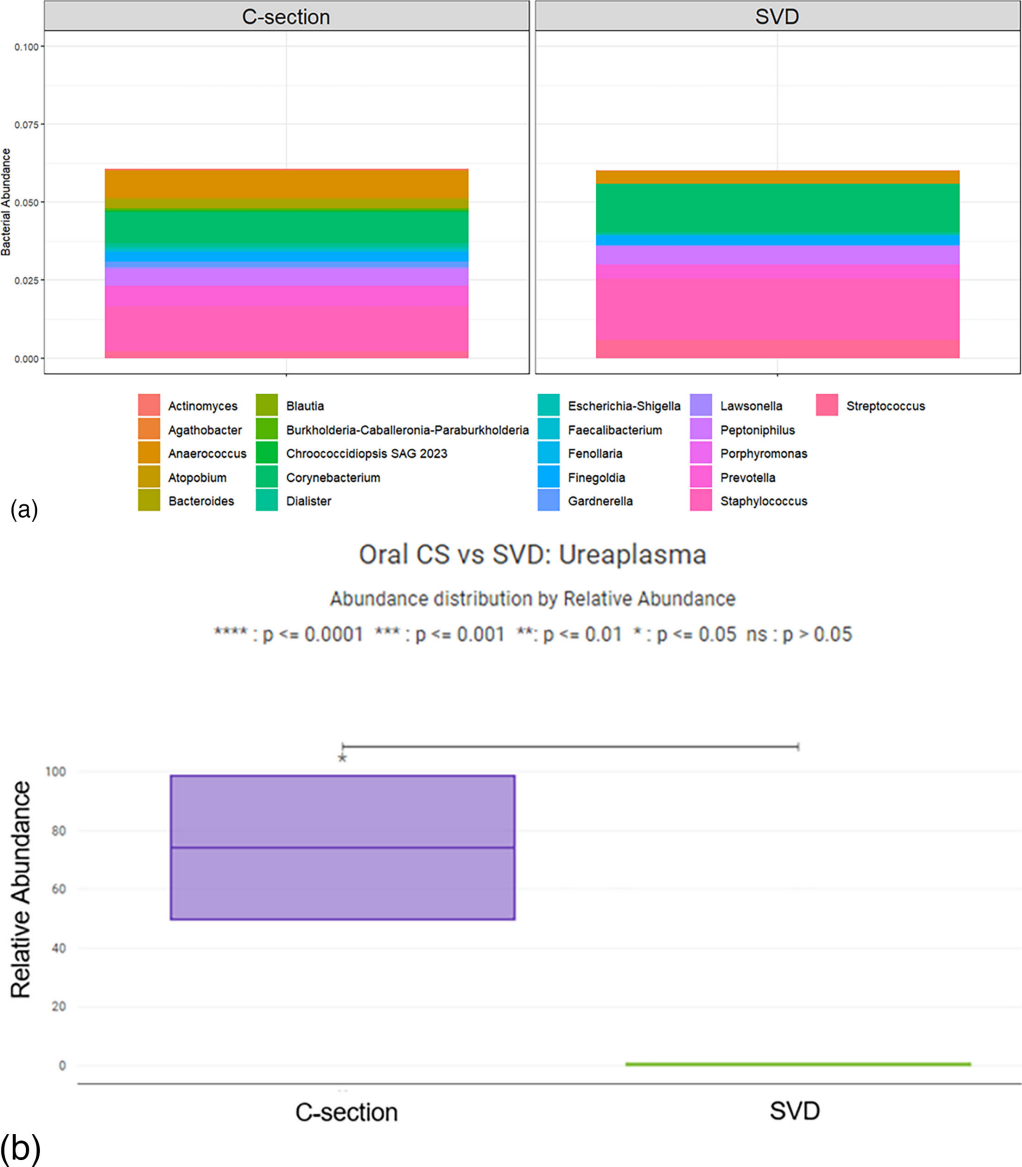
Relative Abundance of Non-*Lactobacillus* Newborn Oral Microbiota by Delivery Method. (a) The average relative abundance of non-*Lactobacillus* genera in the oral microbiota by delivery method at time of delivery. (b) The relative abundance of *Ureaplasma* in the oral cavity of newborns delivered by C-section was significantly higher than those delivered vaginally (*P*=0.046).

When comparing delivery method, the oral microbiome between C-section and vaginally delivered infants was similar (Fig. 5a). There was no significant difference in Chao1 alpha diversity between C-section delivered infants and vaginally delivered infants (*P*=0.865), both with an alpha diversity of 31. C-section delivered newborns had a significantly increased relative abundance of *Ureaplasma* compared to vaginally delivered infants (*P*=0.046) with medians of 74.2% and 0.2%, respectively (Fig. 5b).

### Maternal vaginal and infant microbiome relationships

**Fig. 6.**
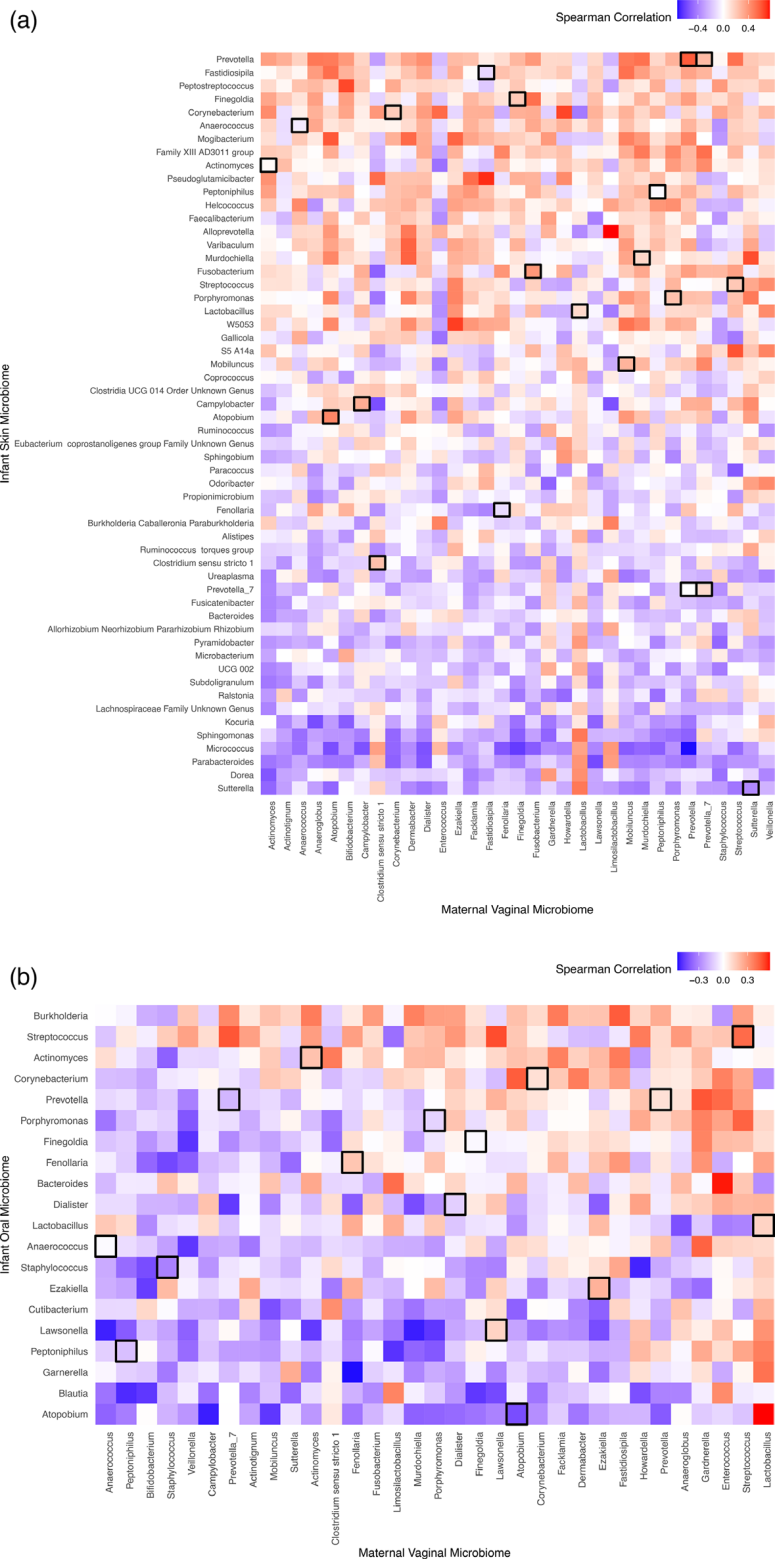
Pairwise Comparisons of Newborn Skin and Oral Microbiomes and Maternal Vaginal Microbiome. (a) Spearman’s Correlation pairwise comparisons of each neonate’s skin microbiome with their respective maternal vaginal microbiome, arranged by the most negative to most positive correlations. Comparisons of the same genus in both maternal and newborn microbiomes are outlined in black. There are several non-significant positive and negative correlations between the mother’s vaginal microbiome and the neonate’s skin microbiome. (b) Spearman’s Correlation pairwise comparisons of each neonate’s oral microbiome with their respective maternal vaginal microbiome, arranged by the most negative to most positive correlations. Comparisons of the same genus in both maternal and newborn microbiomes are outlined in black. There are several non-significant positive and negative correlations between the mother’s vaginal microbiome and neonate’s oral microbiome.

There was no statistically significant correlation between the maternal vaginal microbiome and the skin or oral microbiome of their vaginally delivered newborn. However, there were non-significant positive correlations in most prevalent organisms in both the skin and oral microbiome, such as *Corynebacterium* (rho=0.15)*, Actinomyces* (rho=0.21)*, and Streptococcus* (rho=0.43) in the oral cavity and *Campylobacter* (rho=0.27)*, Finegoldia* (rho=0.27)*, Prevotella* (rho=0.57) and *Porphyromonas* (rho=0.18) on the skin. Upon performing a pairwise Spearman correlation analysis of the neonate’s entire skin microbiome versus the maternal vaginal microbiome, we again found non-significant positive and negative correlations in several organisms. There were negative correlations between *Prevotella* in the mother and *Micrococcus* on the newborn (rho=−0.4). There were positive correlations between *Fastidiosipila* in the mother and *Pseudoglutamicibacter* on the newborn, and *Limosilactobacillus* in the mother and *Alloprevotella* on the newborn (rho >0.4). When correlating the same genus in both the maternal vaginal microbiome and newborn skin microbiome, rho values were near 0 (Fig. 6a). A pairwise Spearman correlation analysis of the neonate’s entire oral microbiome versus the maternal vaginal microbiome also revealed non-significant positive and negative correlations in several organisms. There were negative correlations between *Campylobacter* in the mother and *Atopobium* in the newborn, and *Howardella* in the mother and S*taphylococcus* in the newborn (rho <−0.4). There were positive correlations between *Lactobacillus* in the mother and *Atopobium* in the newborn, and *Enterococcus* in the mother and *Bacteroides* in the newborn (rho >0.4). When correlating the same genus in both the maternal vaginal microbiome and newborn oral microbiome, the majority of rho values were near 0, similar to newborn skin microbiome correlations (Fig. 6b).

## Discussion

The goal of this study was to explore how maternal obesity and mode of delivery contribute to an increased risk of adverse outcomes by influencing oral and skin bacterial dysbiosis in newborns.

### Infant skin microbiome

Higher skin microbiome diversity is associated with skin that is better able to protect against immunological insult [23,24]. Part of a healthy skin microbiota includes *Prevotella*, an anaerobic, Gram-negative commensal bacteria that is found in high numbers in the vaginal flora. Higher abundances of *Prevotella* on infant skin have been positively correlated with decreased severity of atopic dermatitis on the perioral skin of infants [25]. We found that the relative abundance of *Prevotella* on the skin of newborns delivered vaginally was significantly higher than those delivered by C-section. This finding suggests a microbial seeding mechanism by which vaginal delivery could be protective against atopic disease in newborns.

The skin microbiota of C-section delivered neonates was associated with a lower microbial diversity, suggesting the possibility of poorer protection from infection. In our study, C-section delivered neonates had a higher relative abundance of both *Burkholderia-Caballeronia-Paraburkholderia* and *Allorhizobium-Neorhizobium-Pararhizobium-Rhizobium*. Both of these genera are Gram-negative bacilli that are environmental, opportunistic pathogens that can cause serious infections in immunocompromised individuals [26,27]. Additionally, *Burkholderia-Caballeronia-Paraburkholderia* has been positively associated with IgE levels [28], and *Allorhizobium-Neorhizobium-Pararhizobium-Rhizobium* has been identified as a dominant skin genus in plaque psoriasis patients [29]. Collectively, this evidence suggests that the potentially underdeveloped immune system and lower skin bacterial diversity of C-section deliveries may increase the risk of opportunistic infections or atopic disease later in life. Future longitudinal studies are necessary to fully assess this potential risk factor.

When stratified by pre-pregnancy maternal BMI, the relative abundance of neonate skin microbiota was similar; however, there was a higher relative abundance of *Peptoniphilus*, a Gram-positive anaerobic cocci [30], on newborns from obese mothers. A higher relative abundance of vaginal *Peptoniphilus* was also identified in these obese participants, suggesting transfer of these bacteria during pregnancy or delivery [19]. *Peptoniphilus* has been associated with preterm birth, skin infections, chorioamnionitis and neonatal sepsis [28,31]. The higher relative abundance of *Peptoniphilus* found in newborns from obese participants could be another possible mechanism for increased risk of infection or neonatal sepsis for their infants.

### Infant oral microbiome

Newborns born to obese mothers had a higher relative abundance of *Bacteroides* and *Ruminococcus*, two typically commensal residents of the human gut [32,33], in their oral cavities. While these bacteria are commensal when retained in the gut, when found in other body sites, *Bacteroides* can cause bacteremia and abscess formation [33]. Finding higher abundances of *Bacteroides* in the oral cavity of newborns of obese mothers provides yet another possible mechanism for increased infection risk for these newborns. Additionally and controversially, *Bacteroides* exposure in infancy may be related to the risk of obesity later in life [34], suggesting vertical transmission of this bacterial genera through the oral cavity could be a mechanism of obesity heritability in families. Studies have shown that higher abundance of *Ruminococcus* found in the gut can trigger immune cells and lead to the development of respiratory allergies, atopic eczema and inflammatory bowel disease [35,36]. It remains unclear whether a greater abundance of a specific bacteria in the neonate’s mouth directly correlates with higher levels in the digestive tract; however, transfer from the oral cavity to the digestive tract may be one of the initial ways the neonatal gut becomes inoculated. Higher abundance of *Ruminococcus* in the newborn oral cavity could be a mechanism for later pathology.

Previous studies have suggested that vaginally delivered newborns have oral microbiota more similar to their mother’s vaginal microbiota, while C-section delivered newborns have oral microbiota more similar to their mother’s skin microbiome [14]. As such, we expected the vaginally delivered newborns to have lower relative abundances of prevalent skin bacteria such as *Corynebacterium* and higher relative abundance of *Lactobacillus*, but surprisingly, our findings did not reflect this. Upon performing a pairwise analysis of the vaginally delivered neonate’s entire skin and oral microbiomes versus the maternal vaginal microbiome, we found no clear evidence of vertical transfer between mother and vaginally delivered newborn. We found a predominance of *Lactobacillus* in both vaginally delivered and C-section delivered infants with no significant difference in *Corynebacterium* abundance between groups. We found a higher abundance of *Ureaplasma* in the oral cavities of neonates delivered by C-section than those delivered vaginally. *Ureaplasma* are the most prevalent urogenital Mycoplasmataceae [37]. Besides the association with genital tract infections and infertility, *Ureaplasma* has also been shown to colonize amniotic and placental membranes, correlating with preterm birth, newborn infections, chronic lung disease in the newborn and retinopathy of prematurity [38,39]. *Ureaplasma* was present at low levels, with relative abundances less than 3%, in vaginal samples of the participants whose babies were colonized, suggestive of the opportunistic nature of this pathogen and capability of retrograde movement into the uterine space, which was typically considered a sterile site. *Ureaplasma* exposure in C-section delivered neonates may be another mechanism for adverse post-natal outcomes. Our findings in this study bring us closer to understanding the complexities of oral and skin microbiota of newborns and the connection between maternal health-related factors and neonatal outcomes. With over 30% of women of reproductive age being classified as obese, this research is essential for assessing the long-term impact of dysbiosis, mode of delivery and neonatal outcomes. Understanding these relationships will be the cornerstone to developing strategies for preventing adverse neonatal outcomes.

### Limitations

This study did not capture any information about the subsequent changes to the neonatal skin or oral microbiome at the time of delivery or follow infants longitudinally after birth. Our analysis identified differences in both the skin and oral microbiota when stratified by delivery method and maternal pre-pregnancy BMI; however, both the difference in our sample size between vaginal and C-section delivered groups and the total sample size itself prevented a more robust interaction analysis. The neonatal microbiome composition in early life might exert a significant influence on neonatal outcomes. Understanding associations between maternal obesity, delivery method and the relative abundance of certain bacterial genera at the time of delivery may allow us to intervene in the microbial environment to improve newborn outcomes at the time of delivery or even prior to delivery.

### Conclusion/Future directions

We identified differences in the newborn skin and oral microbiomes based on pre-pregnancy maternal BMI and method of delivery. These differences could be linked to an increased risk of allergies, autoimmune disease and infections for neonates. However, we did not find evidence of clear vertical transfer between vaginally delivered infants and the mother’s vaginal microbiome, opening the door for future studies on the impact of the surrounding microbiome in the birthing environment on the newborn microbiome. Future directions for a larger study should gather more data on the target bacterial genera, such as *Peptoniphilus*, *Prevotella* and *Ureaplasma*, and their relationship with neonatal outcomes, including following infants throughout early childhood to determine associations with long-term outcomes. With this deeper understanding, skin and oral microbiota screenings could be used as tools to determine infant risk for post-natal complications, thus allowing for earlier intervention and prevention of poor outcomes.

## Supplementary material

10.1099/jmm.0.002000 Table S1.

10.1099/jmm.0.002000 Table S2.

## Abbreviations

BMI: body mass index
EMR: electronic medical record
IRB: Institutional Review Board
